# Assessing, updating and utilising primary care smoking records for lung cancer screening

**DOI:** 10.1186/s12890-023-02746-4

**Published:** 2023-11-16

**Authors:** Grace McCutchan, Jean Engela-Volker, Philip Anyanwu, Kate Brain, Nicole Abel, Sinan Eccles

**Affiliations:** 1 Division of Population Medicine, School of Medicine, Cardiff University, Cardiff, Wales UK; 2 Academic GP Fellows Scheme, Division of Population Medicine, School of Medicine, Cardiff University, Cardiff, Wales UK; 3 Division of Health Sciences, Warwick Medical School, University of Warwick, Coventry, England UK; 4 Wales Cancer Network, NHS Wales Executive, Cardiff, UK

**Keywords:** Lung cancer, Lung cancer screening, Low-dose CT, Smoking, Electronic healthcare records, Primary care

## Abstract

**Background:**

Lung cancer screening with low-dose computed tomography for high-risk populations is being implemented in the UK. However, inclusive identification and invitation of the high-risk population is a major challenge for equitable lung screening implementation. Primary care electronic health records (EHRs) can be used to identify lung screening-eligible individuals based on age and smoking history, but the quality of EHR smoking data is limited. This study piloted a novel strategy for ascertaining smoking status in primary care and tested EHR search combinations to identify those potentially eligible for lung cancer screening.

**Methods:**

Seven primary care General Practices in South Wales, UK were included. Practice-level data on missing tobacco codes in EHRs were obtained. To update patient EHRs with no tobacco code, we developed and tested an algorithm that sent a text message request to patients via their GP practice to update their smoking status. The patient’s response automatically updated their EHR with the relevant tobacco code. Four search strategies using different combinations of tobacco codes for the age range 55-74_+ 364_ were tested to estimate the likely impact on the potential lung screening-eligible population in Wales. Search strategies included: BROAD (wide range of ever smoking codes); VOLUME (wide range of ever-smoking codes excluding “trivial” former smoking); FOCUSED (cigarette-related tobacco codes only), and RECENT (current smoking within the last 20 years).

**Results:**

Tobacco codes were not recorded for 3.3% of patients (*n* = 724/21,956). Of those with no tobacco code and a validated mobile telephone number (*n* = 333), 55% (*n* = 183) responded via text message with their smoking status. Of the 183 patients who responded, 43.2% (*n* = 79) had a history of smoking and were potentially eligible for lung cancer screening. Applying the BROAD search strategy was projected to result in an additional 148,522 patients eligible to receive an invitation for lung cancer screening when compared to the RECENT strategy.

**Conclusion:**

An automated text message system could be used to improve the completeness of primary care EHR smoking data in preparation for rolling out a national lung cancer screening programme. Varying the search strategy for tobacco codes may have profound implications for the size of the population eligible for lung-screening invitation.

## Introduction

**Fig. 1:**
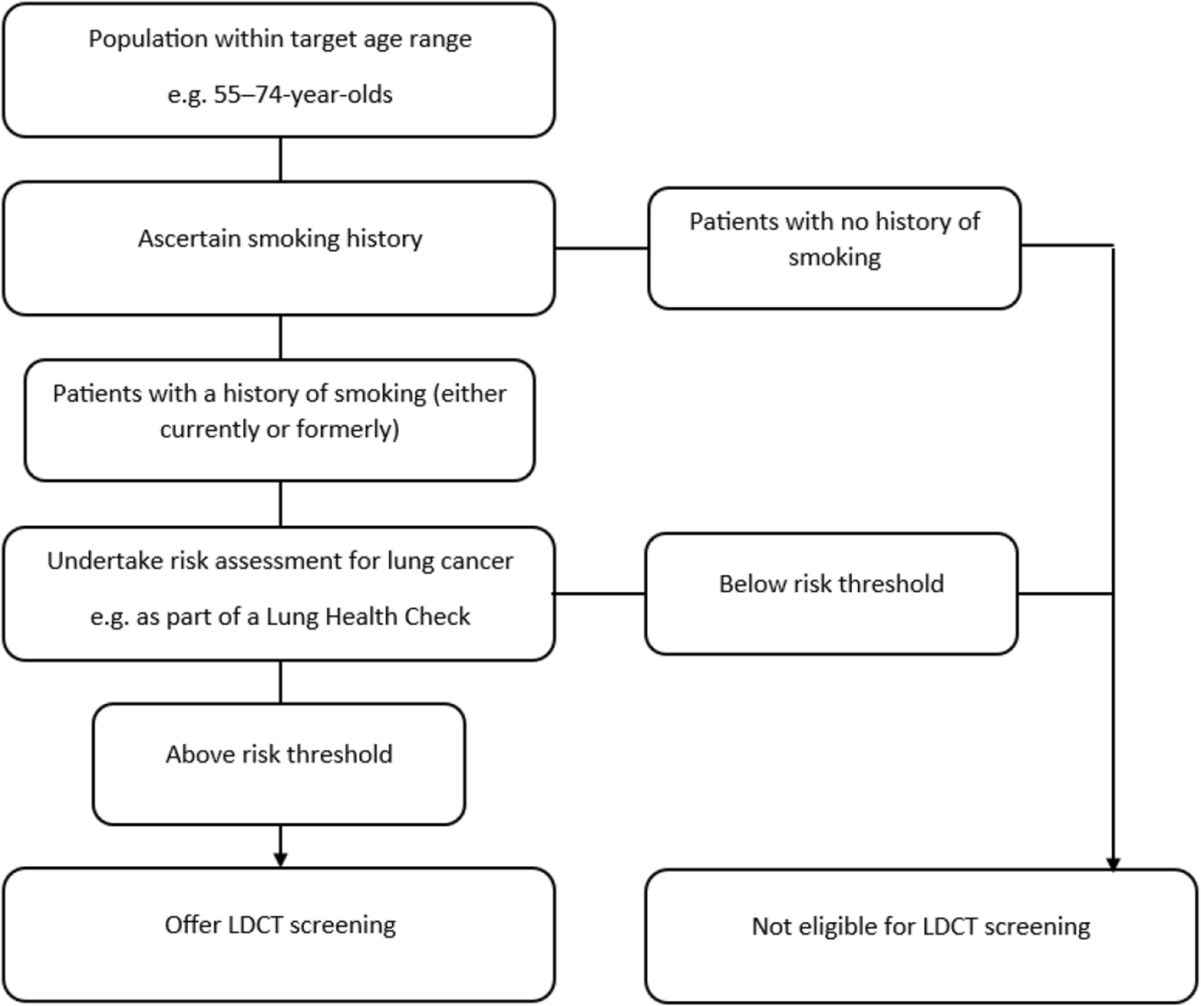
Process to identify high-risk patients for invitation to lung cancer screening

Lung cancer is the leading cause of cancer-related mortality worldwide [1]. Poor outcomes partly reflect high rates of advanced stage diagnoses – currently 71% of cases diagnosed at Stage III/IV in the UK [2] – when curative treatment is rarely possible. Targeted lung cancer screening with low-dose computed-tomography (LDCT) has been shown to reduce mortality by detecting lung cancer at an earlier stage, enabling patients to access a wider range of treatments with curative intent [3–6]. Risk-stratified lung cancer screening based on age and smoking history has been implemented in several high-income countries including the United States [7]. In 2022, the UK National Screening Committee recommended implementation of a national lung cancer screening programme for high-risk individuals aged 55-74 with current or former smoking histories across the four UK nations [8], following the successful Targeted Lung Health Check demonstration pilots in England [9]. Alternative risk-prediction models based on known lung-cancer risk factors such as age, smoking history, occupational exposures and lung comorbidity have been used to assess risk-based eligibility for lung cancer screening via triage Lung Health Check appointment (Fig. 1) [10, 11].

Risk stratification by age and smoking status presents a unique set of challenges for equitable implementation of lung cancer screening, including how best to identify and invite the target population, and planning for capacity/resource based on the number of patients potentially eligible for screening [12, 13]. Resource intensive population-based mailing strategies to invite all age-eligible individuals to book a telephone triage appointment if they have ever-smoked do not yield a good response from individuals who are at highest risk due to current smoking and residence in the most socioeconomically deprived areas [5, 14]. NHS England’s Targeted Lung Health Check standard protocol [15] therefore advises using primary care electronic health records (EHRs) to identify screening-eligible individuals based on age and current/former smoking histories for invitation to a Lung Health Check triage risk assessment to determine eligibility for LDCT lung cancer screening (Fig. 1). However, there are additional challenges associated with utilising EHRs to identify the lung screening-eligible population, including the completeness and accuracy of EHR smoking status records [13, 10] and the breadth of potential EHR tobacco codes that could be used. Research highlights deprivation, age and ethnicity inequalities in completeness and quality of EHR smoking records [16]. Relying on EHRs to identify those eligible for lung cancer screening invitation risks exacerbating existing disparities in screening participation [14, 17–20]. Addressing this issue is a key priority for optimising the clinical and cost-effectiveness of lung cancer screening implementation [12, 13].

Smoking data in primary care EHRs has been found to be of low or moderate accuracy with substantial missing data [10, 21], and existing strategies to update primary care smoking records are resource-intensive [22, 23]. It is therefore essential to assess and improve the quality of primary care smoking data [16] using novel methods [13] to create an inclusive cohort for invitation to a Lung Health Check to assess eligibility for lung cancer screening. In the UK a wide range of tobacco codes can be recorded on EHRs, specifying multiple aspects of the individual’s smoking history [24]. Currently, there is no consensus about which tobacco codes should be used to identify the lung screening-eligible population. One strategy could be to utilise a broad and inclusive range of tobacco codes, but this risks causing harm by over-identifying and inviting patients who are subsequently triaged as ineligible for lung screening [13] and overburdening the system with capacity and resource-related issues [25]. Another approach could be to apply more focused searches for EHR tobacco codes to improve the overall efficiency and cost associated with sending invitations only to those who are highest risk for lung cancer based on recency and heaviness of smoking. Applying focused searches may limit the number of patients invited for triage risk assessment by excluding low-risk individuals, but also inadvertently disregard those who may still be eligible for screening. Therefore, research to test different search strategies for tobacco codes in UK EHRs to identify those potentially eligible for lung cancer screening is required.

The current study involved developing an algorithm and automated text-messaging system to update primary care EHR smoking data. Different combinations of tobacco code search strategies were then tested to estimate the potential size of the population eligible for invitation to a Lung Health Check appointment (Fig. 1).

## Methods

### Study design and aims

This pilot study aimed to (1) examine the completeness of existing EHR tobacco coding to allow identification of those who have ever smoked and would be potentially eligible for a future lung screening programme; (2) develop and test an algorithm to update smoking records in those with no tobacco code recorded using an automated text message system; and (3) test different search strategies using various combinations of tobacco codes to examine the potential effect on the size of the population eligible for an invitation to triage risk assessment to assess eligibility for a lung screening programme in Wales.

### Setting

This study was conducted in Wales, one of the four devolved UK nations with a population of approximately 3.1 million people [26]. Primary care EHRs in Wales are managed using one of two practice management software systems: Vision 360 (“Vision”) or EMIS. UK NICE guidelines state that current smoking or cessation should be assessed by clinicians at every opportunity, and documented within EHRs alongside any details of cessation-related discussions, including support offered and sign-posting [27].

### Participating primary care general practices (GPs)

Seven GP practices in South Wales, with a total patient population of 73,434 as of 2021 (representing 2.3% of the Welsh patient population [26]) were included in this study. All seven GP practices used Vision 360 practice management software and were part of an Academic General Practice (GP) network linked with Academic Fellows in the Division of Population Medicine at Cardiff University.

### Procedures

#### Examining the completeness of existing tobacco codes

All patients with an active and permanent registration status aged 50 to 74_+ 364_ with no “137 Tobacco consumption” code recorded in their EHR were identified using Vision practice management software at the seven included practices. This age range was selected to reflect the age range invited to the NHS England Lung Health Check programme (55-74_+ 364_), with an extension to a lower age group to reflect the population who would enter a rolling programme over the first five years.

#### Testing the algorithm and automated text message system to update EHR smoking records

**Fig. 2:**
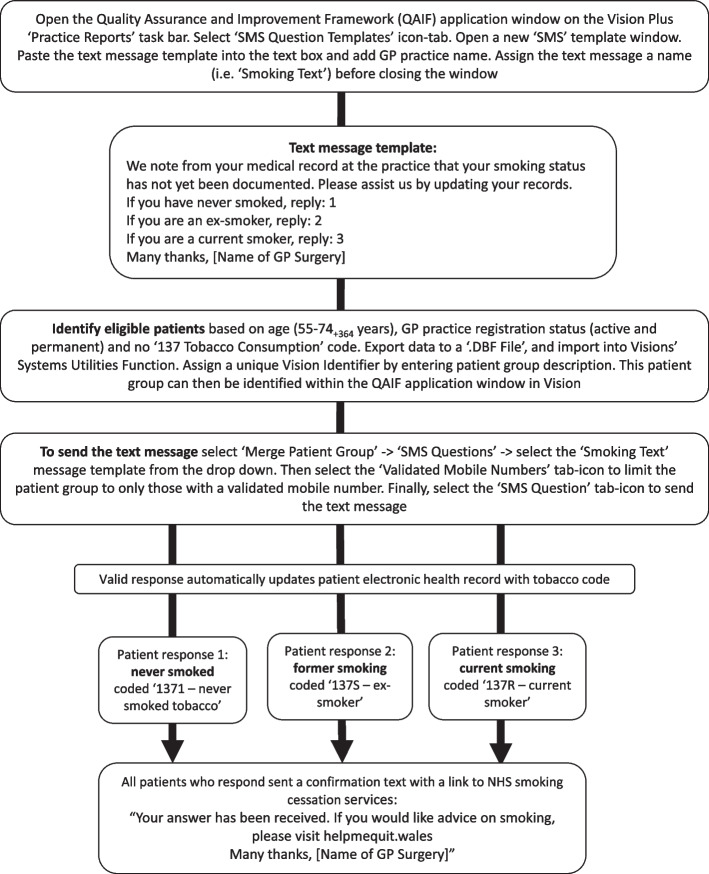
Flow chart of algorithm and automated text-messaging system to update EHR smoking records on Vision Practice Management Software

A text message to request further information from patients about their smoking status was sent to patients with no “137 Tobacco consumption” code using Vision in the seven included practices, Fig. 2. On successful reply, the relevant tobacco code was automatically recorded in the patient record, and a confirmation text message, including a link to the NHS Wales “Help Me Quit” website, was sent to the patient (Fig. 2). Patient records were re-audited between three and 12 days after the smoking status text message was sent to obtain practice-level data on the number of patients receiving and successfully replying with their smoking status.

#### Testing different search strategies for tobacco codes

Searches of the seven GP practice EHRs were run by varying tobacco codes included for patients within the 55-74_+ 364_ age range, with four different tobacco code search strategies developed by the research team (see Table 1). The BROAD search strategy included a wide range of tobacco codes for patients with current/former smoking histories. The VOLUME strategy used the same codes as the BROAD search strategy but excluded the “trivial” former smoking code. The FOCUSSED search strategy used cigarette-related tobacco codes only. The RECENT search strategy included codes for patients who were recorded as currently smoking within the last 20 years.

**Table 1:**
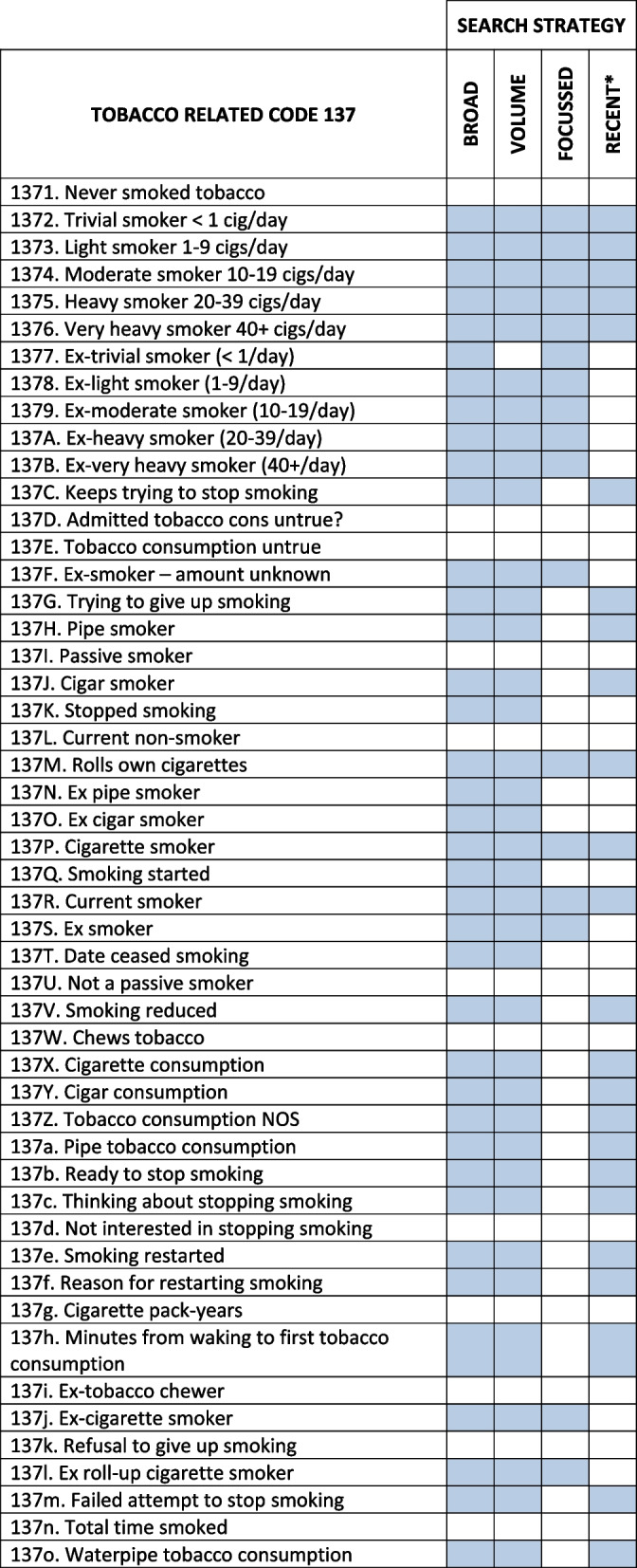
Tobacco codes included in different search strategies

Footnote: blue shaded boxes indicate included codes *The RECENT strategy only included codes recorded within 20 years prior to the search date

### Analysis

For patients with no tobacco code, practice-level data were reported descriptively for the total number of patients (i) with a valid mobile phone number, (ii) who responded to the text message request and (iii) had a history of ever smoking. The total number of patients eligible for lung screening invitation based on the four tobacco code search strategies was reported descriptively for the target age group 55-74_+ 364_, and as a proportion of their age group. The results were then extrapolated from the size of the population included in the study to the population size of Wales to estimate the potential effect on the size of the population eligible for a Lung Health Check.

## Results

### Completeness of smoking records

No tobacco code was recorded in 724/21,956 patients (3.3%) aged 50-74_+ 364_. The percentage of patients aged 50-74_+ 364_ with no tobacco code recorded varied between 0.2-5.3% across included GP practices.

### Testing the algorithm and automated text messaging system to update smoking records

**Fig. 3:**
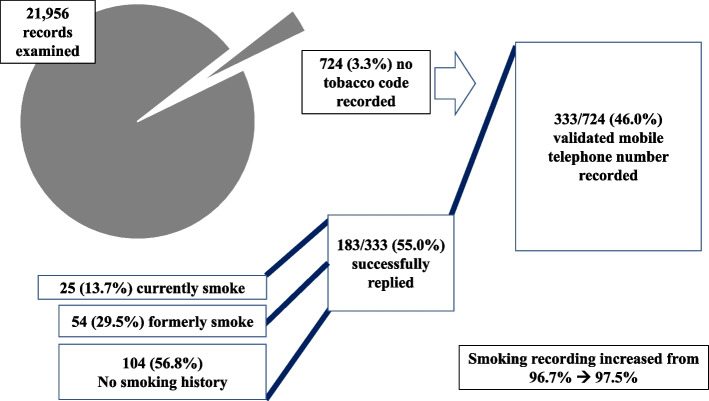
Summary of results from the pilot of a semi-automated text message system to update EHRs with no tobacco code recorded

Of the 724 included patients with no tobacco code recorded, 333 (46.0%) had a validated mobile telephone number within their EHR (Fig. 3). Of these, 183/333 (55.0%) replied to the text message request to update their smoking status with a valid response. Of those who replied, 79/183 (43.2%) indicated that they currently or previously smoked, including 25 who currently smoke (13.7% of replies). Overall, the proportion of patients aged 50-74_+ 364_ with a smoking code recorded increased from 96.7 to 97.5% following the deployment of the text message algorithm.

### Testing different tobacco code search strategies to identify the lung-screening eligible population

Of the 73,434 patients included from the seven practices, 16,916 (23.3%) were in the 55-74_+ 364_ age range. Applying the BROAD, VOLUME and FOCUSSED search strategies resulted in small differences in the number (range: 9090 – 9111) and proportion (range: 53.7-53.9%) of patients eligible for a Lung Health Check within the 55-74_+ 364_ age range (Table 2). Applying the RECENT search strategy reduced the proportion of the patients within the target age range who would be eligible for invitation to a Lung Health Check to 33.7% of those aged between 55 and 74 (Table 2).

**Table 2:** Estimated size of the population eligible for invitation to a Lung Health Check when applying different tobacco code search strategies

Search strategy^a^	Number of eligible patients for invitation within the 55-74_+ 364_ age group for the sample of seven GP practices^b^	Estimated number of eligible patients within Wales for invitation within the 55-74_+ 364_ age group	Estimated % of Welsh population^c^
BROAD	9111	396,130	53.9%
VOLUME	9090	395,217	53.7%
FOCUSSED	9095	395,435	53.8%
RECENT	5695	247,608	33.7%

Footnote: ^a^Please see Table 1 for the full list of included tobacco codes ^b^Total number of patients within the 55-74_+ 364_ age range for the seven include practices, *n* = 16,916 ^c^Estimates for the Welsh population based on the total number of the Welsh population within the 55-74_+ 364_ age range, *n* = 735,478 [20]

When extrapolated to the population size of Wales, the BROAD, VOLUME, FOCUSSED and RECENT strategies would result in an estimated 396,130, 395,217, 395,435 and 247,608 patients aged 55-74_+ 364_ being eligible for invitation to a Lung Health Check, respectively (Table 2). Applying the BROAD search strategy resulted in an additional 148,522 patients eligible to receive an invitation for a Lung Health Check when compared to the RECENT search strategy (37.5% difference).

## Discussion

We piloted a novel method of updating primary care smoking records in preparation for equitable implementation of lung cancer screening delivered via Lung Health Checks in Wales. To our knowledge, this was the first UK study to test a strategy to improve primary care smoking records and examine the use of different search strategies to assess smoking status in EHRs. The completeness of smoking status EHR data was high (over 96%) within the seven GP practices included in the study. Missing smoking record data can be updated using an automated text message system, with almost half (43%) of responders eligible to receive a lung screening invitation using our novel method. Our study suggests that varying the search strategy used could have profound effects on the size and inclusivity of the eligible population for a Lung Health Check, which has potential capacity implications for a national lung cancer screening programme.

Previous UK-based studies have reported similarly high rates of smoking record completeness: 84% [16] and 95% [14]. While emerging evidence highlights efficiency benefits of using targeted approaches to identify and invite patients for a Lung Health Check based on EHR smoking records, modelling shows that relying solely on EHR’s may result in 1.4-2.9% of missed lung cancer diagnoses, highlighting the importance of updating EHR smoking records [14]. The automated text messaging system used in the present study to update smoking records could potentially provide a resource-sparing method to rapidly improve smoking status data completion, particularly in practices with lower data completeness. However, around half of those sent the text message request responded, suggesting that relying solely on this approach may inadvertently exclude patients with missing or out-of-date smoking records. Potential reasons for non-response may include lack of trust or concerns over privacy of data [28] and smoking-related stigma [29], suggesting that multiple approaches may be needed to update EHR smoking data for equitable invitation.

Prior research has demonstrated significant improvements in smoking data capture by using a combination of community outreach methods such as phone calls, text messages, letters and clinician- or nurse-led manual input [22, 23]. Deploying resource-intensive approaches for text message non-responders may be a suitable approach to allay stigma or data-related concerns, and boost response rates to create an inclusive cohort for invitation to screening. Extrapolation from studies of smoking cessation interventions delivered within lung cancer screening suggests that high-intensity interventions are most effective in reaching and engaging under-served populations in smoking cessation (e.g. [30]).

Further research to evaluate this strategy in a larger and more diverse sample of GP practices is warranted, particularly with regards to the potential for digital inequity (i.e. disparities in access/use of mobile phones). Different tobacco code search strategies impacted the potential size of the population for invitation to triage risk assessment to determine eligibility for LDCT screening. While a broad search strategy may be a more inclusive approach to Lung Health Check invitation, it may invite patients who are subsequently found to be ineligible based on risk thresholds, with profound implications for the scale and total cost of the programme. Identifying a higher-risk cohort for invitation (i.e. by recency of smoking using the RECENT search strategy) may result in a greater conversion rate from risk assessment triage to LDCT eligibility, and requires further research to model these effects.

Strengths of our study include the successful development and use of a novel automated text message system to improve the completeness of smoking data in primary care EHRs. Limitations of our study are mostly related to the scale of this pilot. First, we conducted EHR searches in seven primary care practices in South Wales that were part of an academic GP network. These practices may be more ‘research active’ given their link with an academic GP Fellows scheme, which may have influenced the completeness of EHR data. It is possible that the low proportion of missing data in these practices does not represent the pattern of smoking data in non-academic GP practices. Second, examination of the accuracy and completeness of these existing data was beyond the scope of the current study. Discrepancies in self-reported smoking status between different time points, and between self-reported status and recorded status, have been reported in previous studies [31, 32]. While the current pilot study demonstrated the potential of our method to capture missing data on patients without tobacco codes in their primary care records, its application in validating data held for those with a tobacco code is unknown and warrants further research. Third, we developed and tested our algorithm to update smoking data in EHRs on one practice management software system. We are currently developing an algorithm for EMIS - the other primary care practice management software system in use in Wales, and the predominant software system used in England – for testing. Finally, to ensure all extracted data were non-identifiable, practice-level summary data were obtained. However, this strategy precluded individual-level analysis to examine sociodemographic patterning of missing data and text message response. Analysis by patient-level demographics can be used to better direct resource to boost response rates among those with missing data, and should be considered in future research.

## Conclusion

We conducted a pilot of an automated text message system to improve the completeness of smoking status data held in primary care EHRs. Our findings warrant further testing in other GP practices using a variety of software systems in preparation for a national lung cancer screening programme. Using different tobacco code search strategies substantially changed the size of the potential lung screening-eligible population to be invited, and has important implications for the balance of inclusivity, demand and capacity in planning for lung screening implementation.

## Data Availability

All data generated or analysed during this study are included in this published article.

## References

[1] Sung H, Ferlay J, Siegel RL (2021). Global Cancer statistics 2020: GLOBOCAN estimates of incidence and mortality worldwide for 36 cancers in 185 countries. CA Cancer J Clin.

[2] National Lung Cancer Audit. Royal College of Physicians, National Lung Cancer Audit annual report 2022. Available from: [accessed 12.05.2023].

[3] de Koning HJ, van der Aalst CM, de Jong PA (2020). Reduced lung-Cancer mortality with Volume CT screening in a randomized trial. N Engl J Med.

[4] Aberle DR, Adams AM, Berg CD (2011). Reduced lung-cancer mortality with low-dose computed tomographic screening. N Engl J Med.

[5] Field JK, Vulkan D, Davies MPA, et al. Lung cancer mortality reduction by LDCT screening: UKLS randomised trial results and international meta-analysis. The lancet regional health – Europe. 2021;10 10.1016/j.lanepe.2021.100179. 10.1016/j.lanepe.2021.100179 PMC8589726 34806061

[6] Sadate A, Occean BV, Beregi JP (2020). Systematic review and metaanalysis on the impact of lung cancer screening by low-dose computed tomography. Eur J Cancer.

[7] The Lung Cancer Policy Network, 2022. Interactive map of lung cancer screening (first edition). www.lungcancerpolicynetwork.com/interactive-map/.

[8] Lung cancer - UK National Screening Committee (UK NSC) - GOV.UK. https://view-health-screening-recommendations.service.gov.uk/lung-cancer/ (Accessed 12 May 2023).

[9] NHS England. Targeted screening for lung cancer with low radiation dose computed tomography. 2019. https://www.england.nhs.uk/publication/targeted-screening-for-lung-cancer/ (Accessed 12 Apr 2023).

[10] O’Dowd EL, Ten Haaf K, Kaur J (2022). Selection of eligible participants for screening for lung cancer using primary care data. Thorax.

[11] Dickson JL, Hall H, Horst C (2022). Telephone risk-based elebibility assessment for low-dose CT lung cancer screening. Thorax..

[12] Burzic A, O'Dowd EL, Baldwin DR (2022). The future of lung Cancer screening: current challenges and research priorities. Cancer Manag Res.

[13] O'Dowd E, Lee R, Akram A (2023). Defining the road map to a UK national lung cancer screening programme. Lancet Oncol.

[14] Goodley P, Balata H, Alonsoo A, et al. Invitation strategies and participation in a community-based lung cancer screening programme located in areas of high socioeconomic deprivation. Thorax. 2023; 10.1136/thorax-2023-220001. 10.1136/thorax-2023-220001 PMC10803959 37586744

[15] NHS England National Cancer Programme. Targeted Screening for Lung Cancer with Low Radiation Dose Computed Tomography: Standard Protocol prepared for the Targeted Lung Health Checks Programme. 2019. Available at: https://www.england.nhs.uk/wp-content/uploads/2019/02/targeted-lung-health-checks-standard-protocol-v1.pdf (Accessed 12 May 2023).

[16] Dickson J, Hall H, Horst C (2022). Utilisation of primary care electronic patient records for identification and targeted invitation of individuals to a lung cancer screening programme. Lung Cancer.

[17] Baldwin DR, Brain K, Quaife S (2021). Participation in lung cancer screening. Transl Lung Cancer Res.

[18] Lopez-Olivo MA, Maki KG, Choi NJ (2020). Patient adherence to screening for lung cancer in the US. A systematic review and meta-analysis. JAMA Netw Open.

[19] Crosbie PAJ, Gabe R, Simmonds I, et al. Participation in community-based lung cancer screening. ERJ Open. 2022; 10.1183/13993003.00483-2022. 10.1183/13993003.00483-2022 PMC9684623 35777775

[20] Dickson JL, Hall H, Horst C (2023). Uptake of a lung health check invitation offering low-dose CT lung cancer screening to an ethnically and socioeconomically diverse population in a prospective observational cohort study: the SUMMIT study. Lancet Public Health.

[21] Modin HE, Fathi JT, Gilbert CR (2017). Pack-year cigarette smoking history for determination of lung Cancer screening eligibility. Comparison of the electronic medical record versus a shared decision-making conversation. Annals ATS.

[22] Peterson E, Harris K, Farjah F (2021). Improving smoking history documentation in the electronic health record for lung cancer risk assessment and screening in primary care: a case study. Healthc (Amst).

[23] Brenner AT, Cubillos L, Birchard K (2018). Improving the implementation of lung Cancer screening guidelines at an academic primary care practice. J Healthc Qual.

[24] Atkinson MD, Kennedy JI, John A, et al. Development of an algorithm for determining smoking status and behaviour over the life course from UK electronic primary care records. BMC Med Inform Decis Mak. 2017;17(2) 10.1186/s12911-016-0400-6. 10.1186/s12911-016-0400-6 PMC5217540 28056955

[25] Eccles SR, Wright C, Yadollahi R. Lung health check Wales scoping Report. 2020. https://collaborative.nhs.wales/networks/wales-cancer-network/wcn-documents/lung-health-scoping-report/ [accessed 12.05.2023].

[26] Population and household estimates, England and Wales - Office for National Statistics. https://www.ons.gov.uk/peoplepopulationandcommunity/populationandmigration/populationestimates/bulletins/populationandhouseholdestimatesenglandandwales/census2021 (Accessed 10 Oct 2023).

[27] Identifying and quantifying people's smoking - NICE guidelines. In: NICE Recommendations on treating tobacco dependence. Available at: www.nice.org.uk/guidance/ng209/chapter/Recommendations-on-treating-tobacco-dependence#identifying-and-quantifying-peoples-smoking (Accessed 27 Oct 2023).

[28] Belfrage S, Helgesson G, Lynøe N. Trust and digital privacy in healthcare: a cross-sectional descriptive study of trust and attitudes towards uses of electronic health data among the general public in Sweden. BMC Med Ethics. 2022;23(19) 10.1186/s12910-022-00758-z. 10.1186/s12910-022-00758-z PMC8896318 35246118

[29] Quaife SL, Marlow LAV, McEwen A (2017). Attitudes towards lung cancer screening in socioeconomically deprived and heavy smoking communities: informing screening communication. Health Expect.

[30] Williams PJ, Philip KE, Alghamdi SM (2023). Strategies to deliver smoking cessation interventions during targeted lung health screening - a systematic review and meta-analysis. Chron Respir Dis.

[31] Gorber SC, Schofield-Hurwitz S, Hardt J (2009). The accuracy of self-reported smoking: a systematic review of the relationship between self-reported and cotinine-assessed smoking status. Nicotine Tob Res.

[32] West R, Zatonski W, Przewozniak K (2007). Can we trust National Smoking Prevalence Figures? Discrepancies between biochemically assessed and self-reported smoking rates in three countries. Cancer Epidemiol Biomark Prev.

